# Drinking hot beverages is not associated with risk of oesophageal cancers in a Western population

**DOI:** 10.1054/bjoc.2000.1561

**Published:** 2001-01-01

**Authors:** P Terry, J Lagergren, A Wolk, O Nyrén

**Affiliations:** Department of Medical Epidemiology, Karolinska Institutet, Box 281, Stockholm, SE-171 77; Division of Surgery, Karolinska Institutet, Danderyd Hospital, Stockholm, Sweden

**Keywords:** heat, oesophagitis, oesophageal neoplasms, case-control studies

## Abstract

We performed a nationwide population-based case-control study of hot beverage consumption and oesophageal cancer in Sweden. Drinking beverages very hot did not increase the risk for oesophageal squamous cell carcinoma, oesophageal adenocarcinoma, or gastric cardia adenocarcinoma. © 2001 Cancer Research Campaign http://www.bjcancer.com


*British Journal of Cancer* (2001) **84**(1), 120–121
© 2001 Cancer Research Campaign
doi: 10.1054/ bjoc.2000.1561, available online at http://www.idealibrary.com on IDEAL®

http://www.bjcancer.com

Short Communication

### Drinking hot beverages is not associated with risk of oesophageal cancers in a Western population

**P Terry[1], J Lagergren[1,2], A Wolk[1] and O Nyrén[1]**

[1]Department of Medical Epidemiology, Karolinska Institutet, Box 281, SE-171 77 Stockholm; [2]Division of Surgery, Karolinska Institutet, Danderyd Hospital, Stockholm, Sweden

**Summary** We performed a nationwide population-based case-control study of hot beverage consumption and oesophageal cancer in Sweden. Drinking beverages very hot did not increase the risk for oesophageal squamous cell carcinoma, oesophageal adenocarcinoma, or gastric cardia adenocarcinoma. © 2001 Cancer Research Campaign http://www.bjcancer.com

**Keywords**: heat; oesophagitis; oesophageal neoplasms; case-control studies

The association between hot beverages and oesophageal cancer, reported from Asian and South American countries where these beverages are often consumed at practically boiling temperature (Cheng and Day, 1996), is of considerable interest since it hints at a plausible mechanism and since prevention appears to be within reach. Whether drinking hot beverages as they are typically consumed in Western countries is associated with increased risk of oesophageal cancer is, however, unknown. The results of two studies of this issue from Western populations are mixed (Brown et al, 1988; Garidou et al, 1996). Furthermore, previous studies of this issue have mostly examined squamous-cell carcinomas, to the exclusion of adenocarcinomas, which have been rare in the populations under investigation. Several reports of rapid and type-specific increases in the incidence of oesophageal adenocarcinoma, with a risk-factor pattern that is distinct from that in oesophageal squamous cell carcinoma (Lagergren et al, 1999; Cheng et al, 2000), underscore the need for separate analyses by histological type.

We performed a nationwide population-based case-control study of causes of oesophageal cancer in Sweden. The study design has been described in detail elsewhere (Lagergren et al, 1999). In brief, during 1995 through 1997 newly diagnosed patients with adenocarcinoma of the oesophagus (189 cases) or gastric cardia (262 cases) and half of the patients with oesophageal squamous-cell carcinoma (167 cases, born on even dates) in the entire Swedish population below 80 years-of-age were included. Together they represented 81% of all eligible cases, and they were uniformly and thoroughly classified with regard to histologic type and site of the tumour. Control subjects ($n$ = 815) were randomly selected from age and gender strata in the entire Swedish population to mimic the age and gender distribution among cases. Using previously evaluated food frequency questions (Wolk et al, 1997) in computer-aided face-to-face interviews by trained personnel, we asked about usual temperature and frequency of hot beverages consumed 20 years prior to interview in terms of 'cold', 'warm', 'hot', 'very hot', and number of times per day, week, month, or year. There were no subjects who drank neither tea nor coffee. Unconditional logistic regression was used in multivariate modelling, adjusting for potentially confounding factors listed in Table 1. As a basis for the trend tests, scores were constructed from the categorized variables and placed in the model as successive integers.

Drinking beverages very hot did not increase the risk for any of the three studied tumours in age- or multivariate-adjusted models (multivariate results shown in Table 1). On the contrary, it tended to be inversely related to risk, although no significant trends were observed. In analyses restricted to subjects who consumed very

**Table 1** Multivariate-adjusted odds ratios according to beverage temperature

| Temperature of tea and coffee | Adenocarcinoma of oesophagus | Squamous cell carcinoma of oesophagus | Adenocarcenoma of gastric cardia |
|---|---|---|---|
| None, cold, lukewarm | 1.0 (referent) | 1.0 (referent) | 1.0 (referent) |
| Hot | 0.7 (0.5–1.1) | 1.0 (0.6–1.6) | 0.6 (0.4–0.9) |
| Very hot | 0.6 (0.3–1.3) | 0.8 (0.4–1.8) | 0.7 (0.4–1.2) |
| *P* for trend | 0.13 | 0.77 | 0.07 |

Multivariate analyses adjusted for age (5-year age groups), gender, body mass index (quartiles), cigarette smoking (never, past, and current), socioeconomic status (three categories), presence of gastro-oesophageal reflux symptoms (yes or no), frequency quartiles of hot beverage consumption (0–3 per day, 3–5 per day, 5–6 per day, more than 6 per day), and quartiles of alcohol, fruit and vegetables, and energy consumption

*Received 11 August 2000*
*Revised 27 September 2000*
*Accepted 28 September 2000*

*Correspondence to:* P Terry. E-mail: paul.terry@mep.ki.se hot beverages, frequency of intake was not associated with risk (data not shown). In risk-factor models where only beverage temperature (and not frequency of intake) was considered, we also observed no association.

Our large nationwide study is the first to examine the three tumours separately within a single investigation and one of the few to address the issue of hot beverage consumption in Western populations. We found no evidence of any increased risk due to this exposure. Since we asked questions about hot beverage consumption 20 years prior to interview, this result is not likely to be due to individuals with painful tumours avoiding hot beverages. While we cannot rule out a dilution of effect due to non-differential misclassification of exposure, given our results, any increased risk from hot beverage consumption is likely to be small. We conclude that drinking 'hot beverages' by Western standards is not an important public health problem in Western populations and has not contributed to the rising incidence of the adenocarcinomas.

### REFERENCES

Brown LM, Blot W, Shuman S, Smith V, Ershow A, Marks R and Fraumini J (1988) Environmental factors and high risk of oesophageal cancer among men in coastal South Carolina. *J Natl Cancer Inst* **80**: 1620–1625

Cheng K and Day N (1996) Nutrition and esophageal cancer. *Cancer Causes Contr* **7**: 33–40

Cheng K, Sharp L, McKinney P, Logan R, Chilvers C, Cook-Mozaffari P, Ahmed A and Day N (2000) A case-control study of oesophageal adenocarcinoma in women: a preventable disease. *Br J Cancer* **83**: 127–132

Garidou A, Tozonou A, Lipworth L, Signorello L, Kalapothaki V and Trichopoulos D (1996) Life-style factors and medical conditions in relation to oesophageal cancer by histologic type in a low-risk population. *Int J Cancer* **68**: 295–299

Lagergren, J, Bergström R, Lindgren A and Nyren O (1999) Symptomatic gastrooesophageal reflux as a risk factor for oesophageal adenocarinoma. *N Engl J Med* **340**: 825–831

Wolk A, Bergstrom R, Hansson LE and Nyren O (1997) Reliability of retrospective information on diet 20 years ago and consistency of independent measurements of remote adolescent diet. *Nutr Cancer* **29**: 234–241

© 2001 Cancer Research Campaign

